# Residual bone level as a prognostic factor in the surgical treatment of peri-implantitis

**DOI:** 10.3389/fdmed.2024.1532094

**Published:** 2024-12-18

**Authors:** Rodrigo Martin-Cabezas, Catherine Giannopoulou

**Affiliations:** Division of Regenerative Dental Medicine and Periodontology, University Clinics of Dental Medicine, Geneva, Switzerland

**Keywords:** peri-implantitis, marginal bone loss, prognostic factor, surgical peri-implant treatment, treatment outcome, explantation

## Abstract

Peri-implantitis is a progressive inflammatory disease affecting the tissues surrounding dental implants and leading to bone loss. The severity of this disease is typically classified based on the depth of the bone defect or the percentage of bone loss around the implant. Marginal bone loss is a critical factor in the surgical management of peri-implantitis, as it can complicate access for implant decontamination and hinder efforts to stabilize the condition. In cases where bone loss exceeds 50% of the implant length, explantation is often recommended due to significantly reduced success rates after treatment. This narrative review seeks to examine the scientific evidence on marginal bone loss as a prognostic factor in the surgical treatment of peri-implantitis.

## 1 Introduction

Peri-implantitis is an inflammatory disease which affects the tissues surrounding dental implants, characterized clinically by increased probing pocket depth (PPD), bleeding on probing (BOP)/suppuration (SUP) and marginal bone loss (BL) beyond initial bone remodeling (1, 2). The disease progresses rapidly, following a non-linear accelerating pattern (3) that could lead to implant loss (4).

Different approaches for treating peri-implantitis have been proposed including non-surgical and surgical procedures (5). However, implants affected by peri-implantitis are considered to have doubtful prognosis (6) as despite the therapy, 30–50% of treated patients do not achieve complete disease resolution (7). These outcomes seem to be influenced by several factors, such as the implant surface (8), initial BL (9), baseline PPD (10), SUP at baseline (11) level of compliance, plaque control, diagnosis of severe periodontitis (12) and tobacco consumption (13).

The initial BL is related to the magnitude of the lesion. Hence, bone defects can be differentiated according to the BL pattern (intrabony/suprabony), the number of residual bone walls (dehiscence, 2-, 3-walls or circumferential) and the severity of the bone defect (relative BL related to the total implant length or the intra-surgical defect depth in millimeters) (14–16).

**Figure 1:**
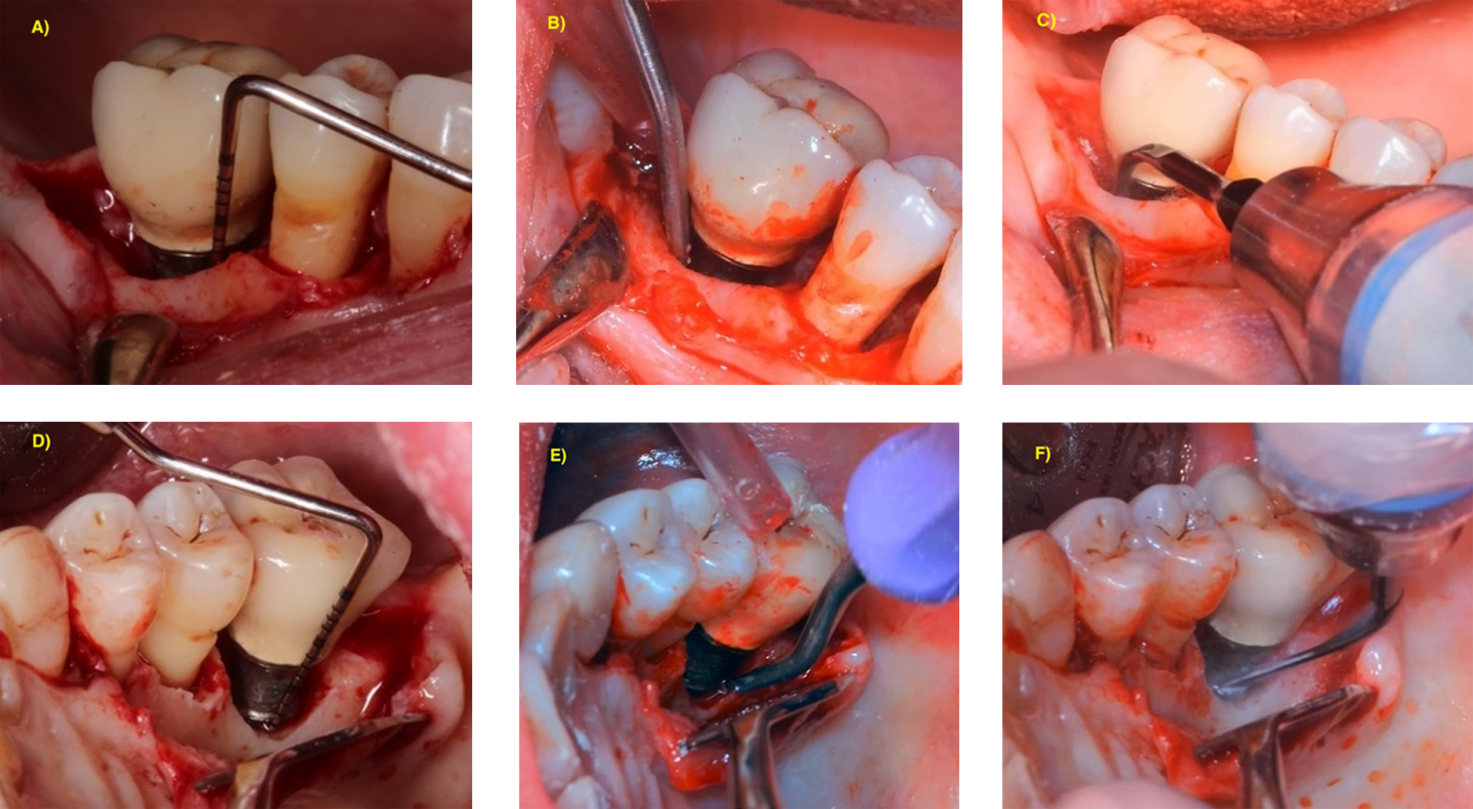
**(A–C)** Access for implant surface decontamination of deep infrabony defect. **(D–F)** Access for implant surface decontamination of deep suprabony defect.

When BL progresses, a significant portion of the implant surface becomes exposed to the oral cavity, making it susceptible to biofilm contamination. Removal of the biofilm can be particularly challenging, especially on implants with rough or modified surfaces (11, 13). In these cases, peri-implant surgery allows better access to the implant surface in order to achieve optimal decontamination (17). However, depending on the amount of initial BL and its morphology, the instrumentation of the implant surface is hindered, with the apical part of the implant becoming less accessible and more difficult to decontaminate (18). Moreover, while suprabony defects allow better instrumentation, intrabony defects could hamper the possibilities for thorough decontamination (19, 20) (Figure 1).

When treating deep defects by resective approaches, the apical repositioning of the flap can be impossible due to the remaining vestibulum height (11). On the other hand, it is not likely that deep bone defects remain self-containing with preservation of the bone walls, which could impact the prognosis for regenerative approaches (21). In addition, BL can result in unfavorable configurations of hard and soft tissues related to the neighboring teeth, that could also hinder optimal cleaning (13). As recently supported by Monje et al. (2023), it can also cause proximal loss of periodontal support for these teeth (22). Finally, after the surgical treatment, a reduced bone level has also been associated with recurrence or progression of the disease (23).

Implant removal can be the most viable option in some severe peri-implantitis cases where the surgical approach has low predictability (24). However, there is no universally accepted threshold beyond which implant preservation becomes impossible (25, 26).

In consequence, the marginal bone level prior to the surgery becomes a key factor in the decision-making process. The aim of this narrative review is to analyze the scientific evidence related to the initial BL in peri-implantitis as prognostic factor prior to surgical treatment.

## 2 Marginal bone loss assessment and classification

Bone remodeling is a physiological process that occurs during the first months of function after prosthesis delivery (2). Historically, different thresholds were proposed to clinically distinguish the physiological bone remodeling from the pathological bone loss (27). Consequently, these differences had affected the reported prevalence of the disease (28). It is accepted that bone remodeling should be limited to 2 mm after prosthesis delivery, and that beyond this threshold, it should be considered as pathological (2). However, these criteria are older and nowadays we should aim to prevent excess remodeling (29).

The marginal bone level is a primary diagnostic factor for peri-implantitis, with a radiographic sensitivity threshold for detecting bone changes of 1.0 mm (30). When initial data is not available to assess the limit of bone remodeling, a 3-mm BL threshold has been proposed for the diagnosis of peri-implantitis in conjunction with the clinical measurements (1, 2).

The long-cone parallel intraoral radiographic projection is recommended for peri-implant bone evaluation which allows to assess crestal bone changes longitudinally (27). The evaluation of marginal BL on radiographs has shown a positive linear correlation between mesial and distal marginal bone levels and the BL evaluated clinically during surgery (30, 31); however, the radiographic measurements resulted frequently in underestimation of the real bone loss assessed after flap elevation (mean 0.7 and 0.6 mm for mesial and distal, respectively) (30).

Although this radiographic technique is considered the gold-standard, it presents an important limitation related to the lack of information on the vestibular and lingual bone walls. When analyzing the residual walls, the morphology of the bone defect was correlated to the buccolingual crestal width, and 4-walls defects were found in broader crests (31). It is important to consider that the vertical component of the BL at buccal and lingual aspects was found to be statistically deeper at 4-walls defects than at 2-walls defects (31). Other authors have analyzed three dimensional assessment of peri-implant BL, however, scatter and artefacts may play a role in reducing the quality of the CBCT imaging, limiting the indication of this radiographic method for the evaluation of peri-implant defects (32).

Based on the literature, multiple classifications have been developed to categorize peri-implant BL (14–16, 33). While Schwarz et al, included only the type of bone loss (suprabony/infrabony) and the residual bone walls (16), other studies proposed the classification of peri-implantitis based on the severity of the BL as follows: Early/Slight, Moderate or Advanced peri-implantitis (BL <25%; 25–50%; >50% of the implant length, respectively) (14, 15, 33). Moreover, the position of the implant in relation to the alveolar crest has recently been included in the classification (BL due to implant malposition or ridge defects). This tridimensional consideration may have prognostic value on determining the best choice of the surgical approach, as for example, regenerative approaches can be limited if implants are placed outside the bone housing (33).

## 3 Initial bone loss and prognosis

Several studies (7–9, 11–13, 34, 35) have analyzed the negative impact of the initial marginal bone levels on the surgical peri-implant outcomes in terms of healing, stabilization or implant failure. All these studies reported an inverse association between the initial severity of the disease and the therapeutic effectiveness. However, the degree of severity is not consistently reported across the studies, and it is classified heterogeneously. Table 1 summarizes the main findings of these studies.

**Table 1:** Studies assessing the association between severity of marginal bone loss and therapeutic outcome.

Study Country	*N*	Type of study	BL classification	Type of surgery	Follow-up	Outcome
Serino & Turri (34), Sweden	31 P/168 I	Prospective case series	2–4 mm	Resective	2 years	Resolution of the disease depended on initial BL. Persistence of the disease is most frequent when initial BL ≥ 5 mm
			5–6 mm			
			>7 mm			
Lagervall & Jansson (12), Sweden	150 P/382 I	Retrospective case series	No BL	Resective or regenerative (xenograft + collagenous membrane)	26 ± 20 months	BL > 1/3 of the length of the implant reduced the effectiveness or the therapy
			BL ≤ 1/3			
			BL > 1/3 ≤ 2/3			
			BL > 2/3			
de Waal et al. (13), Netherlands	74 P/106 I	Retrospective analysis of two RCT	1–3 mm	Resective	1 year	BL at baseline were associated with decreasing success rates
			3–5 mm			
			5–7 mm			
			7–9 mm			
Koldsland et al. (28), Norway	45 P/143 I	Prospective case series	2–3 mm	Resective	6 months	BL > 7 mm was associated with persisting disease (SUP, BOP, PPD > 4 mm + BOP or PPD > 6 mm + BOP)
			>3 mm			
			>5 mm			
			>7 mm			
Serino et al. (7), Sweden	At 2 years: 39 P/208 I	Retrospective case series	NR	Resective	2 years	The effectiveness of the treatment was inversely correlated to the initial bone loss
	At 10 years: 18 P/85 I				10 years	
Lee et al, (35), Korea	45 P/92 I	Retrospective case series	<3 mm	Non-surgical or access flap	6.4 ± 2.7 years	Implants with BL < 3 mm showed better outcome than those with BL ≥ 4 mm. OR = 5.15; 95% CI: 1.20–22.07, *p* = 0.027.
			3 mm			
			≥4 mm			
Ravida et al, (9), USA	80 P/121 I	Retrospective case series	Mild < 25%	Resective or regenerative (allograft and/or xenograft + collagenous membrane)	42.6 ± 26.3 months	BL > 50% length increased 20 time the risk of implant failure, compared to implants with BL < 25%. OR = 20.2; 95% CI: 2.42–169.6; *p* = 0.006.
			Moderate 25%–50%			
			Severe > 50%			
Romandini et al, (8)	149 P/267 I	Retrospective analysis of one RCT and one retrospective case series	BL < 40%	Access flap or resective	7.0 ± 3.6 years	BL ≥ 60% at baseline was a predictor of implant failure
			BL ≥ 40%			
			<60%			
			BL ≥ 60%			

N, population; BL, bone loss; P, participants; I, implants; SUP, suppuration; BOP, bleeding on probing; PPD, probing pocket depth; NR, no reported; OR, odds ratio; CI, confidence interval.

### 3.1 Access flap and resective surgery

Serino et al, conducted a retrospective clinical study on a cohort of patients treated for peri-implantitis by resective surgery for elimination of angular bony defects. The study included implants with BL ≥ 2 mm with a mean BL of 5.5 mm, and single implant restorations were excluded. Subjects were recalled every 6 months for re-evaluation and maintenance care. Sub-gingival scaling at implants presenting with residual pockets was performed during these sessions. At ten-years re-evaluation, 66.6% of implants presenting with deep pockets and inflammation at baseline, remained stable throughout the follow-up period, whereas 29% of the cohort showed further bone loss progression. The authors concluded that the peri-implantitis therapy combined with a high standard of oral hygiene and a 6-month recall program was effective for most of the implants. They also reported that advanced BL before treatment was associated with residual pockets following peri-implant surgery which may be considered a risk indicator for disease progression. As clinical recommendation, authors suggested that the explantation of implants with advanced BL may be the choice of treatment, when a regenerative approach is not feasible and when the prosthetic reconstruction is not compromised. However, it is important to mention that while, this report described “advanced/pronounced/substantial” BL, no threshold was proposed in terms of millimeters or percentage of implant length (7).

The prognostic value of BL in millimeters was evaluated in other studies. In a prospective study including 86 implants treated for peri-implantitis by resective surgery, the outcomes of the therapy were classified according to the initial BL. At 2 years follow-up, 76% of the implants with initial BL between 2 and 4 mm were classified as healthy (PPD < 4 mm without bleeding, suppuration of further BL). In the group with initial BL between 5 and 6 mm, 55% of implants were healthy at the follow-up, while only 22% of implants with an initial BL of ≥7 mm was classified as healthy. Furthermore, explantations were performed exclusively in the group with initial BL of ≥7 mm, with a total of 7 explantations (39%) at 6 months (34). It is important to mention that the treatment of advanced bone defects and deep intrabony defects was limited to bone re-contouring with a resective approach. Thus, for the management of deep intrabony defects, augmentative or combined surgical therapy should be considered (36).

In the same way, the presence of initial BL > 7 mm has been associated to persisting peri-implant inflammation after resective surgery in terms of SUP, BOP or the combination of PPD + BOP (4 mm or 6 mm) (11). In this study with short-term follow-up (6 months), all implants were still present after the therapy. Thus, marginal BL was related to the treatment efficacy, in terms of clinical parameters (SUP, BOP and PPD reduction).

The study of de Waal et al. (2016) was based on two previously conducted randomized controlled trials involving treatment of peri-implantitis with a resective approach. Both trials were conducted by the same group (13). The authors reported that the mean BL at baseline (4.1 ± 1.6 mm) was associated with failure of peri-implantitis treatment after 1 year (OR = 1.46; 95% CI: 1.0–2.1; *p* = 0.043). While implants with a mean initial BL of 1–3 mm showed successful treatment outcome (more than 70% of implants), those with 7–9 mm initial BL showed lower success rates (25% of implants). Among other factors, smoking was associated with poorer treatment outcome. Moreover, the authors highlighted that there is a learning curve for the surgical treatment of peri-implantitis as the amount of experience of the surgical team was significantly associated to treatment success.

It is important to highlight that reporting marginal BL in millimeters has different impact depending also on the initial implant length. In terms of relative BL, Romandini et al. (8), have compared different percentages at baseline as prognostic factors in access flap and resective approaches in the treatment of peri-implantitis. The authors classified relative BL in three categories: <40%; 40–<60%; and ≥60%. Implants with BL ≥ 60% presented the highest risk for implant loss. Interestingly, implants with BL between 40 and 60% did not show significant risk for implant failure compared to implants with BL < 40% (*p* = 0.205).

### 3.2 Reconstructive surgery

For reconstructive surgery, studies analyzed mainly the impact of the defect configuration and residual bone walls on the radiographic bone fill. However, the severity of the defect on treatment effectiveness was not always reported (21). The impact of defect depth has shown controversial results regarding radiographic bone fill. Aghazadeh et al. analyzed the use of resorbable membranes in conjunction with autogenous bone or xenograft, at different types of defect morphology. The authors reported significant radiographic defect fill at the deepest bone defects; a correlation between mesial and distal initial defect depth measured at the time of surgery, and the radiographic outcome was observed (31). On the contrary, another study did not find a correlation between radiographic bone gain and initial marginal bone level (37). However, when analyzing disease resolution with reconstructive surgery in relation to the baseline marginal bone level, slight BL (<25% of the implant length) resulted in better outcomes than the moderate (25%–50% of the implant length) or advanced cases (>50% of the implant length). The success rates were found to be 84.6%, 75% and 71.4%, respectively (37).

The impact of defect morphology has also been analyzed with however conflicting results. While some studies showed that the 4-walls lesion has better reconstructive potential compared to the 2- and 3-walls defects (21, 31), other studies failed to find an association between defect configuration and treatment outcomes (10, 38). Moreover, the defect angle showed a correlation with the radiographic bone gain, with a better defect fill in cases of narrow angles (<40°) (37).

### 3.3 All approaches analyzed together

Some studies pooled together the resective and regenerative approach. Lagervall and Jansson (12) analyzed retrospectively the prognostic factors for treatment success, including non-surgical, surgical resective and regenerative approaches. BL was classified according to the different thirds of the implant length, in degree 1, 2 or 3; severe peri-implantitis was considered when BL was greater than one third of the implant length (degree 2 and 3). Non-surgical therapy was most frequent in patients with BL less than one third of the implant length (degree 1), while defects of degree 2 and 3 were most frequently treated by surgery. The regenerative approach was most frequently performed in severe cases rather than those of degree 1, which could explain the lower success rates of this approach. Explantations were only performed in four patients presenting defects with degree 3. The severity of the lesion reduced the success of the therapy. Implants with BL degree 1 were less prone to have peri-implantitis at the last reevaluation after treatment, compared to those with degree 2 and 3 [Odds Ratio (OR) = 6.5; 95% CI: 1.4–30; *p* < 0.05]. However, degrees 2 and 3 were not analyzed independently, thus not allowing to differentiate between the middle and the apical thirds of the initial BL in terms of prognosis.

Recently, Ravida et al. (9) included also both types of surgery to assess the baseline factors that could influence the outcome. The authors reported that increased BL and implant location (anterior) were associated with implant failure. BL between 25% and 50% of the length of the implant increased the risk of failure by 15 times (OR = 15.2; 95% CI: 2.06–112.7; *p* = 0.008); and BL > 50% increased the risk by 20 times (OR = 20.2; 95% CI: 2.42–169.6; *p* = 0.006). The risk in both cases, was significantly higher compared to implants with BL < 25%. The authors further analyzed independently the two approaches and BL was confirmed to be a prognostic factor for implant failure in both cases. At the end of the study, 39% of implants with >50% of BL were removed. 78.6% did not reach the treatment success criteria (PPD ≤ 5 mm, absence of BOP and bone loss ≤0.5 mm), and only 21% belonging to the group with initial BL of >50% showed clinical signs of health.

## 4 Discussion

The baseline BL prior to treatment can influence the choice of the peri-implant surgical approach (24) and the treatment outcome (13). Several studies have shown low success rates when initial BL is advanced (8, 9, 13). BL is inversely correlated to the effectiveness of therapy: the highest the baseline BL, the lowest the therapeutic success rate, independently of the surgical approach (resective or regenerative) (7–9, 13). Moreover, it has been shown that each additional 1-mm of BL prior to the surgical treatment, increases the risk of future implant failure by 65% (39).

The classification of BL used in the studies is heterogenous, not allowing to establish a cutoff point for explantation. The results of the analyzed studies depend on the reference point for the statistics and conclusions should be interpreted carefully. Thus, when the reference point was stated at BL < 40, the statistic threshold for implant failure was BL > 60% (8); however, when the reference was < 25%, the risk for failure increased statistically even for BL 25%–50% (9). Similar results occurred when the reference point was 33% of the implant length, with higher risk for BL up to the middle third (12). The differences between studies can be in part due to the disparities on “advanced BL” definition. Thus, it is important to report consistently the severity of the lesion, keeping the term “advanced” for BL > 50% (14, 15, 33).

When BL is restricted to one third of the implant length, non-surgical therapy may, in some cases, resolve the disease without further need of surgical treatment (12); however, surgical approaches in those situations, have shown better outcomes with success rates up to 70% (13, 34, 37). More factors other than apical BL extent such as defect configuration or soft tissue quality may influence the therapeutic choice and outcome (9, 31, 39).

It is recognized that for the treatment of the majority of advanced peri-implant lesions, surgical approaches are often needed (40). However, when the marginal BL reaches the apical region of the implant, the prognosis becomes hopeless and explantation should be considered (6). In fact, the stabilization of implants with BL > two thirds of the length is unpredictable (8).

From a clinical point of view, the diagnosis of peri-implantitis when baseline data is not available requires a PD ≥ 6 mm + BOP/SUP + marginal BL ≥ 3 mm (1). Considering a standard implant (10 mm length), BL would be almost at the middle third. When the implant BL affects the middle third of the implant (33%–66%), the decision between peri-implant surgery or explantation becomes more sensitive. When BL is in the middle third, studies have shown worst prognosis compared to those presenting BL < 25%–33% of the length (9, 12), however another study did not found significant differences between BL < 60% and BL < 40% (8). Thresholds of BL > 50%–60% of the implant appear to be aligned with the clinical reality. Furthermore, a study analyzing at which BL level implants affected by peri-implantitis were explanted, concluded that the practitioners performed explantations when implants presented a mean BL of 66.5% (25).

**Figure 2:**
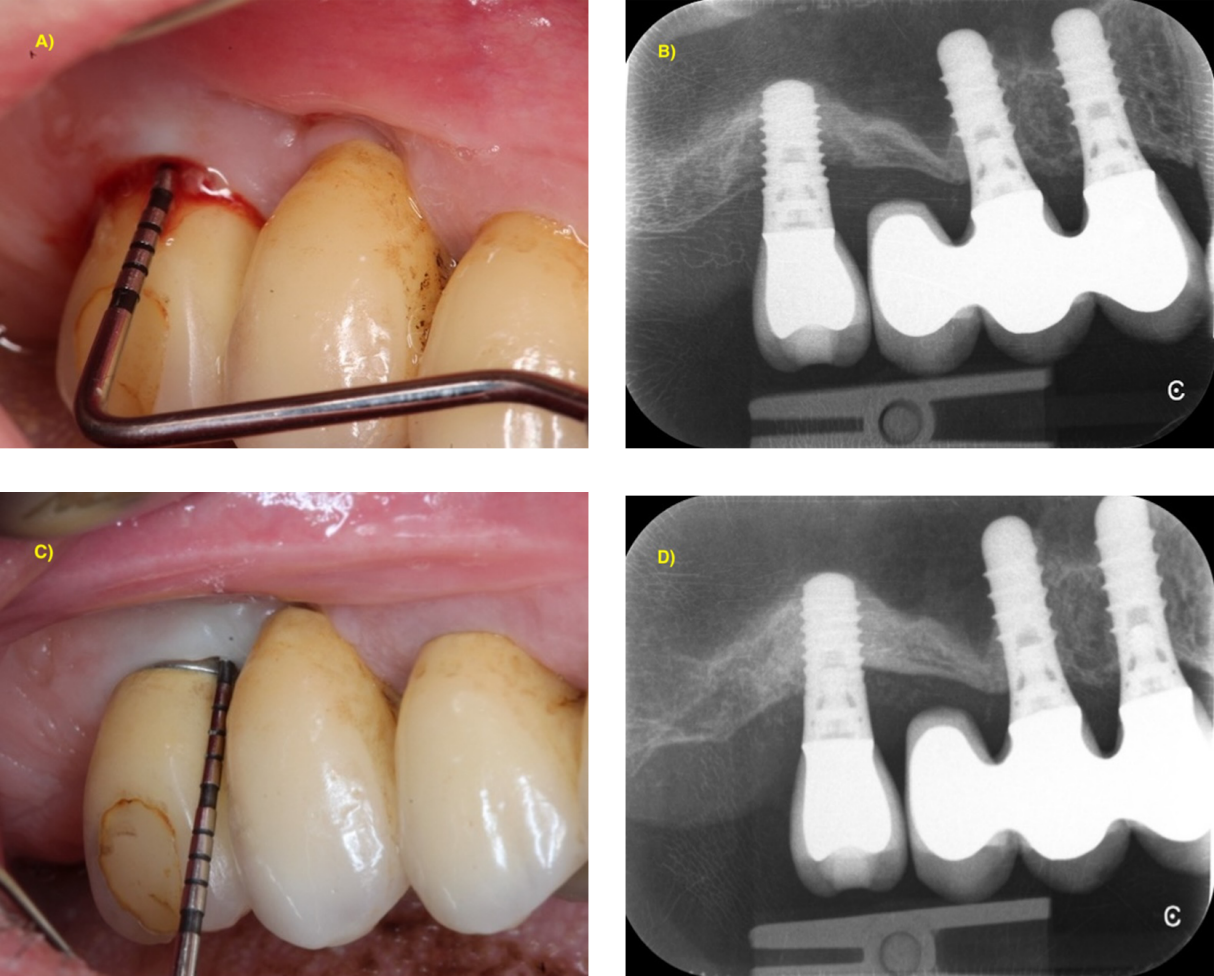
1-year stabilization of advanced peri-implantitis showing BL > 50% of implant length with combined surgical approach (reconstructive + implantoplasty). **(A)** Baseline clinical situation with PPD = 8 mm and BOP; **(B)** periapical x-ray showing intrabony bone loss up to the apical third of the implant; **(C)** clinical situation at 1-year post-operative follow-up with PPD = 4 mm without BOP; **(D)** periapical x-ray showing complete bone filling of the intrabony component.

In this context, BL exceeding 50% of the implant length has been proposed as threshold for explantation (4, 24). However, the stabilization of implants with advanced BL is also achievable, thus explantation based only on bone levels could result in the loss of opportunity for some patients to maintain their implants (Figure 2). Moreover, although the success rates of implants placed in early failed implants sites are high (96% compared to 98% for implants placed for the first time), the new implants had significantly higher risk of failure (41). Hence, “rescue therapy” can be performed in advanced cases, depending also on patient desires (24) as the surgical treatment performed in implants with BL > 50% has shown 21% success rate (9), thus avoiding the sequela from implant explantation (42). In those cases a thorough examination of the risk indicators is important for establishing a pretherapeutic prognosis, such as hard and soft tissue deficiencies, tobacco consumption, medical condition, implant surface or cleanability of the prosthetic restoration (6, 8, 13). In fact, smoking is associated to advanced BL (13), residual pockets after surgical peri-implant treatment, and is considered as a prognostic indicator in treatment outcome (13). Thus, smoking cessation is advised in both pre- and post-surgical care (10). In addition, technical factors that may influence our treatment choice, such as the risk of potential damage of the neighboring anatomical structures (maxillary sinus or inferior alveolar nerve) or the prosthetic implication of the implant, should be considered.

Further research for development and validation of composite models for peri-implant prognosis is needed. These models should combine several risk factors to allow the practitioner to decide the best option according to the risk classification. In our knowledge, only one model is nowadays available based on Nobel Biocare implants (43). This model considers that BL of more than one third of implant length (>33%) and combined with factors, such as history of periodontitis, early disease development (<4 years of function) and implant length >13 mm, as having unfavorable prognosis. Unfortunately, this model cannot distinguish finer degrees of BL, highlighting the need for further research to support practitioners in making evidence-based decisions regarding explantation or peri-implant surgery.

In conclusion, several limiting factors pertain to the early diagnosis of peri-implantitis and its accuracy, such as the low sensitivity of intra-oral radiographs for detecting bone changes of approximately 1.0 mm (30), the low sensitivity of peri-implantitis classification for early-stage detection of the disease in the absence of baseline data (44), the rapid progression of the disease (3), and the prognostic significance of initial marginal BL (13). This situation highlights the importance of baseline documentation following implant placement for the early diagnosis and treatment of peri-implantitis.

## 5 Conclusion

BL is a key pretherapeutic prognostic factor for peri-implant surgical treatment outcome, as treatment effectiveness is inversely correlated with initial bone levels. In advanced cases, where BL exceeds 50–60% the success rates decrease significantly, thus the decision for explantation should be considered.

## References

[1] Berglundh T Armitage G Araujo MG Avila-Ortiz G Blanco J Camargo PM Peri-implant diseases and conditions: consensus report of workgroup 4 of the 2017 world workshop on the classification of periodontal and peri-implant diseases and conditions. J Periodontol. (2018) 89(Suppl 1):S313–S8. 10.1111/jcpe.12957 29926955

[2] Renvert S Persson GR Pirih FQ Camargo PM. Peri-implant health, peri-implant mucositis, and peri-implantitis: case definitions and diagnostic considerations. J Periodontol. (2018) 89(Suppl 1):S304–S12. 10.1002/JPER.17-0588 29926953

[3] Derks J Schaller D Hakansson J Wennstrom JL Tomasi C Berglundh T. Peri-implantitis—onset and pattern of progression. J Clin Periodontol. (2016) 43(4):383–8. 10.1111/jcpe.12535 26900869

[4] Monje A Nart J. Management and sequelae of dental implant removal. Periodontol 2000. (2022) 88(1):182–200. 10.1111/prd.12418 35103326

[5] Herrera D Berglundh T Schwarz F Chapple I Jepsen S Sculean A Prevention and treatment of peri-implant diseases-the EFP S3 level clinical practice guideline. J Clin Periodontol. (2023) 50(Suppl 26):4–76. 10.1111/jcpe.13823 37271498

[6] Orishko A Imber JC Roccuzzo A Stahli A Salvi GE. Tooth- and implant-related prognostic factors in treatment planning. Periodontol 2000. (2024) 95(1):102–28. 10.1111/prd.12597 39234949

[7] Serino G Wada M Mameno T Renvert S. Two- and ten-year follow-up of patients responding and non-responding to the surgical treatment of peri-implantitis: a retrospective evaluation. Clin Oral Implants Res. (2021) 32(4):410–21. 10.1111/clr.13711 33449388

[8] Romandini M Bougas K Alibegovic L Hosseini S Carcuac O Berglundh T Long-term outcomes and prognostic factors of surgical treatment of peri-implantitis—a retrospective study. Clin Oral Implants Res. (2024) 35(3):321–9. 10.1111/clr.14228 38112108

[9] Ravida A Siqueira R Di Gianfilippo R Kaur G Giannobile A Galindo-Moreno P Prognostic factors associated with implant loss, disease progression or favorable outcomes after peri-implantitis surgical therapy. Clin Implant Dent Relat Res. (2022) 24(2):222–32. 10.1111/cid.13074 35320880

[10] Ichioka Y Trullenque-Eriksson A Ortiz-Vigon A Guerrero A Donati M Bressan E Factors influencing outcomes of surgical therapy of peri-implantitis: a secondary analysis of 1-year results from a randomized clinical study. J Clin Periodontol. (2023) 50(10):1282–304. 10.1111/jcpe.13848 37461197

[11] Koldsland OC Wohlfahrt JC Aass AM. Surgical treatment of peri-implantitis: prognostic indicators of short-term results. J Clin Periodontol. (2018) 45(1):100–13. 10.1111/jcpe.12816 28902415

[12] Lagervall M Jansson LE. Treatment outcome in patients with peri-implantitis in a periodontal clinic: a retrospective study. J Periodontol. (2013) 84(10):1365–73. 10.1902/jop.2012.120555 23237584

[13] de Waal YC Raghoebar GM Meijer HJ Winkel EG van Winkelhoff AJ. Prognostic indicators for surgical peri-implantitis treatment. Clin Oral Implants Res. (2016) 27(12):1485–91. 10.1111/clr.12584 25818042

[14] Monje A Pons R Insua A Nart J Wang HL Schwarz F. Morphology and severity of peri-implantitis bone defects. Clin Implant Dent Relat Res. (2019) 21(4):635–43. 10.1111/cid.12791 31087457

[15] Froum SJ Rosen PS. A proposed classification for peri-implantitis. Int J Periodontics Restorative Dent. (2012) 32(5):533–40. 22754901

[16] Schwarz F Herten M Sager M Bieling K Sculean A Becker J. Comparison of naturally occurring and ligature-induced peri-implantitis bone defects in humans and dogs. Clin Oral Implants Res. (2007) 18(2):161–70. 10.1111/j.1600-0501.2006.01320.x 17348880

[17] Schwarz F Jepsen S Obreja K Galarraga-Vinueza ME Ramanauskaite A. Surgical therapy of peri-implantitis. Periodontol 2000. (2022) 88(1):145–81. 10.1111/prd.12417 35103328

[18] Steiger-Ronay V Merlini A Wiedemeier DB Schmidlin PR Attin T Sahrmann P. Location of unaccessible implant surface areas during debridement in simulated peri-implantitis therapy. BMC Oral Health. (2017) 17(1):137. 10.1186/s12903-017-0428-8 29183313 PMC5706147

[19] Keim D Nickles K Dannewitz B Ratka C Eickholz P Petsos H. *In vitro* efficacy of three different implant surface decontamination methods in three different defect configurations. Clin Oral Implants Res. (2019) 30(6):550–8. 10.1111/clr.13441 31009116

[20] Luengo F Sanz-Esporrin J Noguerol F Sanz-Martin I Sanz-Sanchez I Sanz M. *In vitro* effect of different implant decontamination methods in three intraosseous defect configurations. Clin Oral Implants Res. (2022) 33(11):1087–97. 10.1111/clr.13991 35997508 PMC9825956

[21] Schwarz F Sahm N Schwarz K Becker J. Impact of defect configuration on the clinical outcome following surgical regenerative therapy of peri-implantitis. J Clin Periodontol. (2010) 37(5):449–55. 10.1111/j.1600-051X.2010.01540.x 20374416

[22] Monje A Pons R Amerio E Lin GH Ortiz-Gonzalez L Kan JY Proximal periodontal support adjacent to untreated peri-implantitis lesions: a cross-sectional analysis. Int J Oral Maxillofac Implants. (2023) 38(6):1145–50. 10.11607/jomi.10415 38085745

[23] Carcuac O Derks J Abrahamsson I Wennström JL Berglundh T. Risk for recurrence of disease following surgical therapy of peri-implantitis-A prospective longitudinal study. Clin Oral Implan Res. (2020) 31(11):1072–7. 10.1111/clr.13653 32870513

[24] Sinjab K Garaicoa-Pazmino C Wang HL. Decision making for management of periimplant diseases. Implant Dent. (2018) 27(3):276–81. 10.1097/ID.0000000000000775 29762186

[25] Wentorp F Jablonowski L Pink C Holtfreter B Kocher T. At which bone level are implants explanted? Clin Oral Implants Res. (2021) 32(7):786–98. 10.1111/clr.13747 33755997

[26] Solderer A Al-Jazrawi A Sahrmann P Jung R Attin T Schmidlin PR. Removal of failed dental implants revisited: questions and answers. Clin Exp Dent Res. (2019) 5(6):712–24. 10.1002/cre2.234 31890309 PMC6934347

[27] Sanz M Chapple IL, Working Group 4 of the VEWoP. Clinical research on peri-implant diseases: consensus report of working group 4. J Clin Periodontol. (2012) 39(Suppl 12):202–6. 10.1111/j.1600-051X.2011.01837.x 22533957

[28] Koldsland OC Scheie AA Aass AM. Prevalence of peri-implantitis related to severity of the disease with different degrees of bone loss. J Periodontol. (2010) 81(2):231–8. 10.1902/jop.2009.090269 20151801

[29] Linkevicius T Apse P Grybauskas S Puisys A. The influence of soft tissue thickness on crestal bone changes around implants: a 1-year prospective controlled clinical trial. Int J Oral Maxillofac Implants. (2009) 24(4):712–9. 19885413

[30] Serino G Sato H Holmes P Turri A. Intra-surgical vs. radiographic bone level assessments in measuring peri-implant bone loss. Clin Oral Implants Res. (2017) 28(11):1396–400. 10.1111/clr.12999 28009061

[31] Aghazadeh A Persson RG Renvert S. Impact of bone defect morphology on the outcome of reconstructive treatment of peri-implantitis. Int J Implant Dent. (2020) 6(1):33. 10.1186/s40729-020-00219-5 32548733 PMC7297900

[32] Kuhl S Zurcher S Zitzmann NU Filippi A Payer M Dagassan-Berndt D. Detection of peri-implant bone defects with different radiographic techniques—a human cadaver study. Clin Oral Implants Res. (2016) 27(5):529–34. 10.1111/clr.12619 26059443

[33] Rosen PS Froum SJ Sarmiento H Wadhawani CP. A revised peri-implantitis classification scheme: adding three-dimensional considerations to facilitate prognosis and treatment planning. Int J Periodontics Restorative Dent. (2022) 42(3):291–9. 10.11607/prd.5876 35472104

[34] Serino G Turri A. Outcome of surgical treatment of peri-implantitis: results from a 2-year prospective clinical study in humans. Clin Oral Implants Res. (2011) 22(11):1214–20. 10.1111/j.1600-0501.2010.02098.x 21309860

[35] Lee SB Lee BA Choi SH Kim YT. Long-term outcomes after peri-implantitis treatment and their influencing factors: a retrospective study. J Periodontal Implant Sci. (2022) 52(3):194–205. 10.5051/jpis.2103020151 35775695 PMC9253284

[36] Ramanauskaite A Cafferata EA Begic A Schwarz F. Surgical interventions for the treatment of peri-implantitis. Clin Implant Dent Relat Res. (2023) 25(4):682–95. 10.1111/cid.13162 36419243

[37] Monje A Pons R Sculean A Nart J Wang HL. Defect angle as prognostic indicator in the reconstructive therapy of peri-implantitis. Clin Implant Dent Relat Res. (2023) 25(6):992–9. 10.1111/cid.13244 37405662

[38] Roccuzzo M Gaudioso L Lungo M Dalmasso P. Surgical therapy of single peri-implantitis intrabony defects, by means of deproteinized bovine bone mineral with 10% collagen. J Clin Periodontol. (2016) 43(3):311–8. 10.1111/jcpe.12516 26800389

[39] Ravidà A Saleh I Siqueira R Garaicoa-Pazmiño C Saleh MHA Monje A Influence of keratinized mucosa on the surgical therapeutical outcomes of peri-implantitis. J Clin Periodontol. (2020) 47(4):529–39. 10.1111/jcpe.13250 31912526

[40] Renvert S Polyzois I. Treatment of pathologic peri-implant pockets. Periodontol 2000. (2018) 76(1):180–90. 10.1111/prd.12149 29239086

[41] Chatzopoulos GS Wolff LF. Survival rate of implants performed at sites of previously failed implants and factors associated with failure: a retrospective investigation. J Dent Sci. (2024) 19(3):1741–7. 10.1016/j.jds.2023.10.012 39035295 PMC11259635

[42] Gargallo-Albiol J Tavelli L Barootchi S Monje A Wang HL. Clinical sequelae and patients’ perception of dental implant removal: a cross-sectional study. J Periodontol. (2021) 92(6):823–32. 10.1002/JPER.20-0259 32997346

[43] Nobre MA Salvado F Nogueira P Rocha E Ilg P Malo P. A prognostic model for the outcome of nobel biocare dental implants with peri-implant disease after one year. J Clin Med. (2019) 8:1352. 10.3390/jcm8091352 PMC6780417 31480537

[44] Romandini M Berglundh J Derks J Sanz M Berglundh T. Diagnosis of peri-implantitis in the absence of baseline data: a diagnostic accuracy study. Clin Oral Implants Res. (2021) 32(3):297–313. 10.1111/clr.13700 33340418

